# Sinularin, an Anti-Cancer Agent Causing Mitochondria-Modulated Apoptosis and Cytoskeleton Disruption in Human Hepatocellular Carcinoma

**DOI:** 10.3390/ijms22083946

**Published:** 2021-04-11

**Authors:** Chou-Yuan Ko, Po-Chang Shih, Po-Wei Huang, Yi-Hsin Lee, Yen-Fu Chen, Ming-Hong Tai, Chi-Hao Liu, Zhi-Hong Wen, Hsiao-Mei Kuo

**Affiliations:** 1 Division of Gastroenterology and Hepatology, Department of Internal Medicine, Kaohsiung Armed Forces General Hospital, Kaohsiung 80284, Taiwan; gastroenterokjy@gmail.com (C.-Y.K.); lineageofray@hotmail.com (Y.-F.C.); 2 Institute of Medical Science and Technology, National Sun Yat-sen University, Kaohsiung 80424, Taiwan; 3 Department of Marine Biotechnology and Resources, National Sun Yat-sen University, Kaohsiung 80424, Taiwan; po-chang.shih.14@ucl.ac.uk (P.-C.S.); smoke88722@gmail.com (P.-W.H.); lesinsin@gmail.com (Y.-H.L.); 4 UCL School of Pharmacy, University College London, Bloomsbury, London WC1N 1AX, UK; 5 Institute of Biomedical Sciences, National Sun Yat-sen University, Kaohsiung 80424, Taiwan; minghongtai@gmail.com; 6 Center for Neuroscience, National Sun Yat-sen University, Kaohsiung 80424, Taiwan; 7 Division of Nephrology, Department of Interanl Medicine, Kashsiung Armed Forces General Hospital, Kaohsiung 80284, Taiwan; colinliu1201@gmail.com

**Keywords:** sinularin, mitochondria, apoptosis, reactive oxygen species, hepatocellular carcinoma

## Abstract

Liver cancer remains a leading cause of death, despite advances in anti-cancer therapies. To develop novel drugs, natural products are being considered as a good source for exploration. In this study, a natural product isolated from a soft coral was applied to evaluate its anti-cancer activities in hepatocellular carcinoma SK-HEP-1 cells. Sinularin was determined to have half-maximal inhibitory concentration (IC_50_) values of ~10 μM after 24, 48, and 72 h. The TUNEL assay and annexin V/PI staining results showed that sinularin induced DNA fragmentation and apoptosis, respectively. An investigation at the molecular level demonstrated that the expression levels of cleaved caspases 3/9 were significantly elevated at 10 μM sinularin. Mitochondrial and intracellular reactive oxygen species (ROS) levels were significantly increased following sinularin treatment, which also affected the mitochondrial membrane potential. In addition, it significantly lowered the mitochondrial respiration parameters and extracellular acidification rates at 10 μM. Further investigation showed that sinularin significantly attenuated wound healing, cell migration, and potential colony formation at 10 μM. Fluorescence microscopic observations showed that the distribution of F-actin filaments was significantly altered at 10 μM sinularin. Supported by Western blot analyses, the expression levels of AKT, p-ERK (extracellular-signal-related kinase), vimentin and VEGF were significantly down-regulated, whereas p-p38, pJNK and E-cadherin were significantly increased. Overall, at the IC_50_ concentration, sinularin was able to significantly affect SK-HEP-1 cells.

## 1. Introduction

Globally, liver cancer is the most common cause of death among cancer diseases, and it is the only cancer having an annual increase in the occurrence rate among the top five deadliest cancer types [1]. The incidence rate of hepatocarcinoma varies across the globe, with the greatest prevalence in East Asia and the fifth-highest prevalence in the United States. Equally, the prevalent risk factors for liver cancer vary by country [2]. Commonly known risk factors include hepatitis B virus, hepatitis C virus, fatty liver disease, alcohol-related cirrhosis, and diabetes [3]. Affected patients are often diagnosed at later stages, which leads to a poor prognosis. Currently, only 5–15% of patients with hepatic carcinomas are eligible for surgical resection. This is due to the fact that surgical removal is only applicable to early-stage patients and that hepatic regenerative capacity is ablated at the later stages. More than 90% of liver cancer cases are categorized as hepatocellular carcinomas (HCC), which are best treated with chemotherapy and immunotherapy [4]. In spite of advances in treatment modality, HCC remains elusive [5]. Therefore, research efforts on anti-HCC drug development are intensively ongoing.

Cancer progression involves a process of tumorigenesis (i.e., formation of tumors that are related to apoptosis/cell survival and angiogenesis), immune evasion, and metastasis. Apoptosis is a specific form of cell death that is tightly regulated in normal cells. It is involved in normal cell development, aging, immune reactions under pathological threats, and chemical-induced cell death, etc. Commonly known characteristics of apoptosis include DNA fragmentation, activation of cysteine-dependent aspartate-specific proteases (caspases) 3/9, and, morphologically speaking, cellular shrinkage [6]. Apoptosis is also referred to as programmed cell death; however, it becomes dysregulated in cancer cells, which can lead to the formation of a tumor mass. Without an adequate blood supply, it is believed that the tumor mass will not grow beyond 0.2 cm in diameter [7]. Accordingly, angiogenesis is required for tumor growth, and vascular endothelial growth factor (VEGF) is a well-known regulator of blood vessel formation. Prior to progressing to immune invasion, cancer cells need to be mobile and migrating to dissociate from the primary tumor mass. Molecularly speaking, cell migration can be regulated by mitogen-activated protein kinases (MAPKs), including Jun N-terminus kinase (JNK), p38, and ERK (extracellular-signal-related kinase). To promote cell migration, these kinases are able to coordinate complex signaling pathways to distinctively activate one or multiple signaling axes in stimulus- and cell type-dependent manners [8]. Cell migration can also be regulated by epithelial-mesenchymal transition (EMT), which causes the loss of cell–cell junctions and dissociates cells from each other, thus increasing cell mobility [9]. Intriguingly, E-cadherin and vimentin, which are biomarkers of EMT, can be modulated by MAPKs [10,11,12]. Upon completion of the immune invasion, cancer cells are able to metastasize around the body to establish secondary tumors in any organ, resulting in difficulty in treating cancer.

Mitochondria are organelles endowed with opposite roles in the cell. They can generate adenosine triphosphate (ATP) for the regulation of cell metabolism and survival. On the contrary, mitochondria also act as cell death effectors that induce an intrinsic apoptosis pathway [13]. ATP is known to be mainly produced by oxidative phosphorylation (OXPHOS) occurring on the inner mitochondrial membrane in the cell. The electron transfer chain (ETC) of the OXPHOS contains proton pumps that convert oxidizable substrates to generate electron flow across the pumps and translocate hydrogen ions from the mitochondrial matrix to the intermembrane space. The proton pumping leads to a proton gradient across the inner mitochondrial membrane, which drives protons back into the mitochondrial matrix via ATP synthase, which simultaneously converts adenosine diphosphate (ADP) into ATP. The OXPHOS is highly regulated as high-energy electrons pass along the proton pumps of the ETC. However, under stressed conditions, it can be a reactive oxygen species (ROS) generator that ultimately induces an intrinsic apoptosis pathway. Intriguingly, mitochondria are also linked to migration modulation and metastasis through, for instance, ROS-induced regulation of MAPKs phosphorylation or pathways associated with the EMT [14]. Collectively, these suggest that mitochondria can be considered as good targets to attenuate cancer progression.

An arsenal of natural products has been explored for development as anti-neoplastic therapies, and natural products extracted from sea creatures have become an alternative source to land creatures. Marine compounds derived from soft corals, a type of sea animal, have long been believed to yield promising drug candidates. Sinularin, first extracted from the soft coral *Sinularia flexibilis,* was collected from Hayman Island on the Great Barrier Reef of Australia [15]. This compound is also referred to as flexibilide [16]. After intensive studies, sinularin has been found to be active against inflammation [17], cardiovascular disease [18], and microbial infection [19]. More intriguingly, this marine compound has also shown activities against cancers such as epidermoid carcinoma [15], renal cancer [20], breast cancer [21], gastric carcinoma [22], melanoma [23], oral cancer [24], and HCC (HepG2 and Hep3B cells) [25]. Although the activities of sinularin against HCC cell lines HepG2 and Hep3B have been reported, its activity against and mechanism on the HCC cell line SK-HEP-1 remain unknown. In the study using HepG2 cells, Chung et al. reported the effects of siunularin on DNA damage, cell cycle arrest, and mitochondrial membrane potential; however, they did not demonstrate the results of mitochondria dysfunction and -associated proteins to support their claims [25]. In our study, sinularin was subjected to SK-HEP-1 cells to assess the effect of cell mitochondria and molecular functions during apoptosis. Further investigation included cell migration, colony formation, and cytoskeleton distribution in parallel with the analysis of critical proteins that are considered important in the regulation of the above cellular functions. Overall, in comparison with the report of Chung et al., our work provides more supportive results showing anti-cancer effects of sinularin on HCC cells.

## 2. Results

### 2.1. Sinularin Significantly Induced Apoptosis through Caspase-Activated DNA Fragmentation

**Figure 1:**
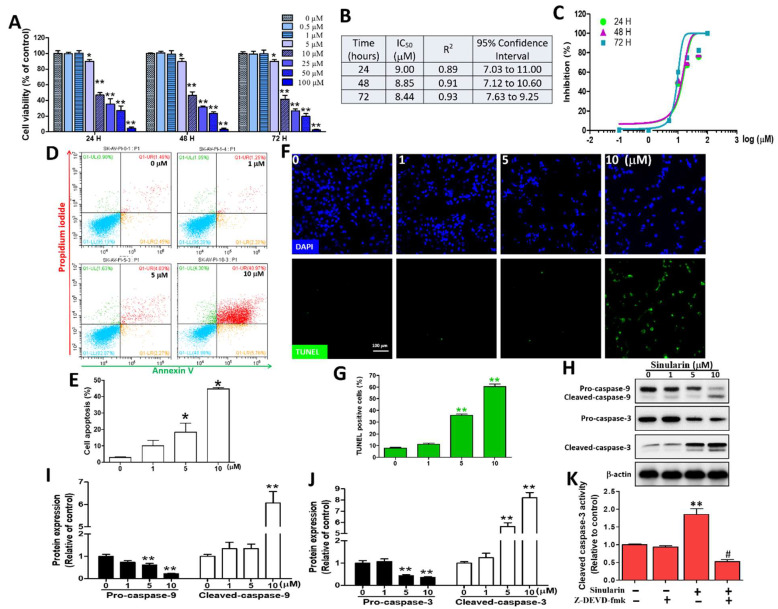
The sinularin induces cell viability, DNA fragmentation, and caspases 3/9 in SK-HEP-1 cells. (**A**) Quantification of cell viability using the MTT (3-(4,5-Dimethylthiazol-2-yl)-2,5-diphenyltetrazolium bromide) assay with sinularin at concentrations of 0, 0.5, 1, 5, 10, 25, 50, or 100 μM for 24, 48, and 72 h, with results expressed as the percentage of cell viability compared to untreated cells. (**B**) Determination of the IC_50_ values of sinularin after 24, 48, and 72 h. R^2^, representing coefficient of determination, and 95% confidence interval values were calculated. (**C**) Concentration-response S curves for sinularin after 24, 48, and 72 h of incubation. The cell proliferation inhibition response (*y*-axis), and logarithmic concentration (x-axis) in the presence of different sinularin concentrations. R^2^ and 95% confidence interval values of each concentration curve are shown in Figure 1B. (**D**) Flow cytometric analyses of apoptosis using Annexin-V/PI staining in the cells treated with sinularin after 24 h. The dot-plot quadrant diagram reflects the Annexin-V (x-axis; green) and PI (y-axis; red). The lower left quadrant shows the result for normal cells (Annexin V−/PI−) and the upper left quadrant shows the result for necrotic cells (Annexin V−/PI+), while the lower right quadrant shows the result for early apoptotic cells (Annexin V+/PI−) and the upper right quadrant shows the result for late apoptotic cells (Annexin V+/PI+). (**E**) Quantification of the Annexin V+/PI− (early apoptotic cells) and Annexin V+/PI+ (late apoptotic cells) regions. (**F**) Immunofluorescence showing apoptotic bodies in the SK-HEP-1 cells (marked in green) by the TUNEL-BrdU assay after being treated with 0, 1, 5, or 10 μM of sinularin for 24 h. DAPI staining was used to observe cellular DNA/nuclei (blue) and was visualized under a fluorescence microscope (200× magnification). (**G**) Quantification of TUNEL-positive cells. (**H**) A representative Western blot showing bands of caspases 3/9 in their pro- and cleavage forms, together with the housekeeper protein β-actin after being treated with sinularin for 24 h. Full, uncropped Western blot gels are shown in Supplementary Figure S2A. (**I**) Pro- and cleaved caspase 9 levels quantified with densitometry analysis using ImageJ software after normalized with the β-actin level. (**J**) The pro- and cleaved caspase 3 levels quantified with densitometry analysis using ImageJ software after being normalized with the β-actin level. (**K**) After prior incubation with Z-DEVD-fmk (5 μM) for 2 h, SK-HEP-1 cells were treated without or with sinularin (10 μM) for an additional 24 h and then examined by cleaved caspase-3 kit and ELISA read analysis. Each bar represents the mean ± SE of three independent experiments. The results were analyzed using Student’s *t*-test and ANOVA to determine the significance. * *p* < 0.05 and ** *p* < 0.01 relative to the control (sinularin-untreated cells), and ^#^ *p* < 0.01 relative to the experimental group with 10 μM of sinularin alone.

To understand cell viability under sinularin treatments, a range of concentrations (0, 0.5, 1, 5, 10, 25, 50, or 100 μM) was evaluated in SK-HEP-1 cells using the MTT (3-(4,5-dimethylthiazol-2-yl)-2,5-diphenyltetrazolium bromide) assay. After 24 h of incubation, the percentage of cell viability under 0, 0.5, 1, 5, 10, 25, 50, and 100 μM of sinularin was 100.00 ± 9.02, 99.90 ± 1.70, 100.00 ± 3.12, 90.15 ± 2.07, 46.89 ± 2.91, 35.36 ± 6.99, 26.96 ± 6.07 and 4.25 ± 1.67%, respectively. We also evaluated the same sinularin concentrations for 48 and 72 h. The half-maximal inhibitory concentration (IC_50_) of sinularin was determined to be ~9.0, 8.8, and 8.5 μM after 24, 48, and 72 h, respectively (Figure 1A–C). Therefore, we applied 10 μM (~IC_50_ value) or a lower range of 0–10 μM to study the effect of sinularin. To assess the mode of cell death induced by sinularin, Annexin V and PI-stained cells were subjected to flow cytometric analyses. It was reported that Annexin V+/PI− indicated an early apoptotic cell population (the lower right quadrant), while Annexin V+/PI+ indicated a late apoptotic cell population (the upper right quadrant) [13]. Figure 1D shows the typical transfer of apoptotic cells (early and late) from the left quadrant to the right quadrant in SK-Hep-1 cells treated with 0, 1, 5, and 10 μM of sinularin for 24 h. The findings showed that the numbers of apoptotic cells were significantly increased to 18.43 ± 5.42% and 44.74 ± 0.72% at 5 and 10 μM of sinularin, relative to the control (2.98 ± 0.41%, 0 μM of sinularin, Figure 1E). At a magnification of 200X, the TUNEL assay and immunofluorescence staining of the SK-HEP-1 cells after treatment with 0, 1, 5, and 10 μM of sinularin showed DNA fragmentation (upper, green fluorescence). All nuclei (lower, blue fluorescence) were stained with DAPI (4′,6-diamidino-2- phenylindole) (Figure 1F). The results showed that TUNEL-positive cells induced the DNA fragmentation of SK-HEP-1 cells in a concentration-dependent manner under 5 and 10 μM of sinularin, which significantly increased to 35.72 ± 1.08% and 60.39 ± 2.15%, relative to the control (7.91 ± 0.46%, 0 μM sinularin, Figure 1G). Subsequently, Western blot analyses were carried out to understand the expression profiles of pro-caspases-3/9 and cleaved-caspases-3/9, with all proteins normalized with β-actin (Figure 1H). Sinularin treatment between 0–10 μM decreased the amount of pro-caspases-9 (an approximate 80% decrement) and pro-caspases-3 (an approximate 65% decrement), which was followed by a significant increase of the cleaved-caspase-9 form (about a 6.1-fold increment) and the cleaved-caspase-3 form (about an 8-fold increment), leading to apoptosis (Figure 1I,J). Following pre-treatment with and without 10 μM of a caspase-3 inhibitor (Z-DEVD-fmk), the caspase-3 activity was significantly reversed compared to sinularin alone (Figure 1K). In addition to evaluating cell viability using SK-HEP-1 cells, we determined the IC_50_ values in another HCC cell line (Huh-7) and in a normal mouse liver cell line (Clone 9), at ~20 and >100 μM, respectively, following sinularin treatments for 24, 48, and 72 h (Supplementary Figure S1). Taken together, the IC_50_ of the sinularin for normal liver cancer cells was more than 5-fold the dosage of the HCC cells, while the results of the cell viability, annexin-V/PI staining, TUNEL immunofluorescence, and Western bottling showed that sinularin treatment could significantly enhance the apoptosis of SK-HEP-1 cells.

### 2.2. Production Levels of Intracellular and Mitochondrial ROS (mtROS) Were Augmented, While the Mitochondrial Membrane Potential (ΔΨ) Was Reduced after Sinularin Treatments

**Figure 2:**
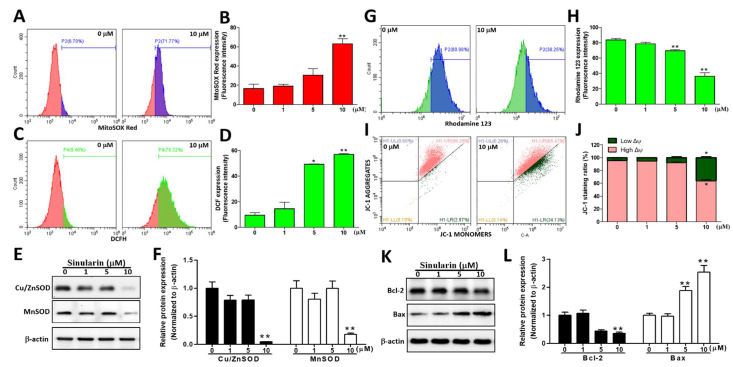
Sinularin induced the mitochondrial O^2–^ and intracellular ROS production, the loss of mitochondrial membrane potential, and expression of relevant proteins in SK-HEP-1 cells. (**A**) The fluorescence intensity of mitochondrial O^2–^, measured using MitoSOX^TM^ Red under 0 or 10 µM of sinularin for 24 h. (**B**) Quantification of O^2–^ accumulation in the mitochondria through analyzing the gated range of single-parameter histograms from 5 × 10^3^–10^6^. (**C**) The fluorescence intensity of intracellular ROS, measured using CM-H_2_DCFDA under 0 or 10 µM of sinularin for 24 h. (**D**) Quantification of DCF (2′,7′-dichlorofluorescein) signals from the intracellular ROS level via analyzing the gated range of single-parameter histograms from 5 × 10^3^–10^6^. (**E**) SK-HEP-1 cells treated with different concentrations of sinularin for 24 h. The cell lysates were subjected to Western blot analysis of the bands of Cu/ZnSOD, MnSOD, and the internal control β-actin. Full, uncropped Western blot gels are shown in Supplementary Figure S2B. (**F**) Cu/ZnSOD and MnSOD protein levels, quantified by densitometry analysis using ImageJ software after being normalized with the β-actin level. (**G**) SK-HEP-1 cells treated with and without 10 μM of sinularin for 24 h. ΔΨ depolarization effects were detected using cell-permeable cationic Rhodamine 123 and flow cytometry. (**H**) Quantification of Rhodamine 123 signals. The quantitative values were obtained after analyzing the gated range of single-parameter histograms from 2 × 10^6^–10^7^. (**I**) SK-HEP-1 cells were treated with and without 10 μM of sinularin for 24 h. ΔΨ depolarization effects were detected using cell-permeable cationic JC-1 and flow cytometry, and mitochondrial depolarization was indicated by a decrease in the red fluorescence signal. (**J**) Quantification of JC-1 signals. The quantitative values were obtained after analyzing the gated range of the quadrant plot from high ΔΨ (upper right) and low ΔΨ (lower right). (**K**) Western blot analysis with antibodies against Bcl-2, Bax, and the internal control β-actin. Full, uncropped Western blot gels are shown in Supplementary Figure S2C. (**L**) Bcl-2 and Bax protein levels, quantified by densitometry analysis using ImageJ software after being normalized with the β-actin level. Each bar represents the mean ± SE of three independent experiments. The results were analyzed using Student’s *t*-test to determine the significance, in which * *p* < 0.05 and ** *p* < 0.01 relative to the control (sinularin-untreated cells).

Due to high-energy electrons in the OXPHOS, which can result in electron leaks, mitochondria are assumed to be the main generators of ROS in a cell. ROS, such as O^2–^ and H_2_O_2_, can activate caspase-mediated DNA breakage. Hence, a CM-H_2_DCFDA fluorescent probe was employed to detect ROS levels intracellularly, in parallel with the MitoSOX^TM^ Red fluorescent probe to detect mitochondrial O^2–^ levels, following 24 h of sinularin treatments at 0, 1, 5, or 10 μM. Our results showed that both intracellular ROS and mitochondrial O^2–^ levels were elevated with the increasing concentrations of sinularin. The MitoSOX^TM^ Red signals were significantly increased to 63.13 ± 5.14% at 10 μM, relative to the control (16.51 ± 4.45%, Figure 2A,B). Likewise, the fluorescence levels of DCF (the product of hydrolysis and oxidation of CM-H_2_DCFDA) were significantly elevated to 49.17 ± 0.27 and 56.84 ± 0.55% at 5 and 10 μM, respectively, relative to the control (9.40 ± 2.00%, Figure 2C,D). We subsequently monitored the expression levels of superoxide dismutase, such as Cu/ZnSOD and MnSOD, which are known to be involved in the transformation of oxygen free radicals into hydrogen peroxide and oxygen molecules [26]. Our findings showed that the expression levels of Cu/ZnSOD and MnSOD were significantly ablated following 10 μM of sinularin treatment (Figure 2E,F). To understand the impact of sinularin on mitochondrial membrane potential (ΔΨ), 10 μM of either Rhodamine 123 or JC-1 dyes were added. The cationic dye Rhodamine 123 can specifically bind to the mitochondrial membrane and can emit fluorescence under cytofluorometry [27]. In healthy cells, the positively charged JC-1 can enter into mitochondria and form irreversible J-aggregates that emit red fluorescence (~590 nm). In contrast, in unhealthy or apoptosis cells, J-aggregates are not preferentially formed, and thus JC-1 original green fluorescence (~529 nm) remains [28]. The results demonstrated that the fluorescence intensity of Rhodamine 123 was significantly decreased at 10 μM of sinularin, indicating that ΔΨ was also significantly decreased. At the same sinularin concentration, the ratio of low ΔΨ/high ΔΨ was found to be significantly increased, suggesting a de-energized ΔΨ (Figure 2G–J). We also monitored the expression of the apoptosis suppressor Bcl-2 and a pro-apoptotic protein Bcl-2 associated X protein (Bax). Our results showed that at 10 μM of sinularin treatment, the Bcl-2 expression level was significantly abated, while that of Bax was significantly increased (Figure 2K,L). These results suggested that sinularin could enhance cellular and mtROS through the inhibition of the antioxidant enzymes in HCC cells while reducing the mitochondrial membrane potential, resulting in a significant outspread of the Bcl-2 protein family, including pro-apoptotic molecules and anti-apoptotic molecules.

### 2.3. Effect of Sinularin on the Oxygen Consumption Rate, Extracellular Acidification Rate, and ETC Complex I–V Proteins of Mitochondria in SK-HEP-1 Cells

**Figure 3:**
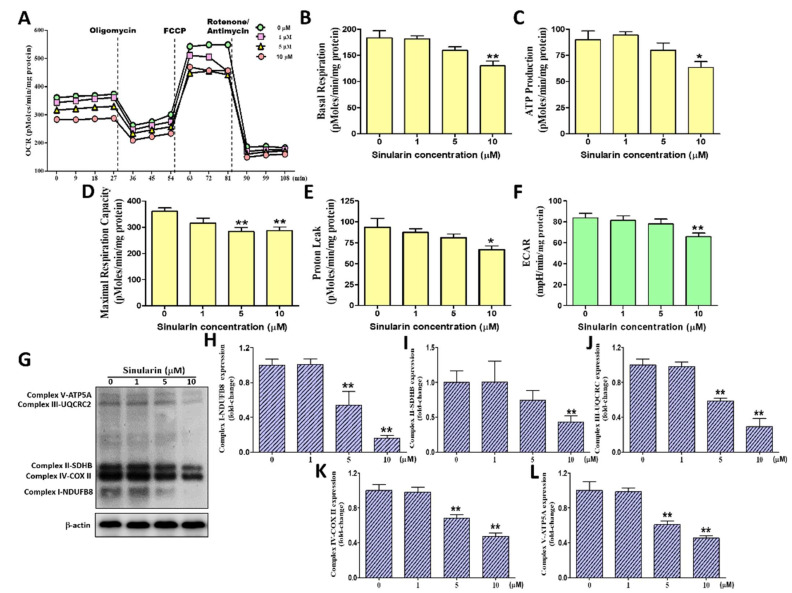
The effects of sinularin on the OCRs (Oxygen consumption rates) parameter, ECARs (extracellular acidification rate), and 5 OXPHOS (Oxidative phosphorylation) enzymatic complexes in SK-HEP-1 cells. The OCRs (pMoles/min/mg protein) were measured before and after the addition of pharmacological agents to living cells. The ECARs (mpH/min/mg protein) were measured before the addition of pharmacological agents to living cells. Four measurements were taken and averaged to provide reliable data as a base value, followed by sequential and continuous injections of Seahorse XF Cell Mito Stress Test reagents, including oligomycin, FCCP, and antimycin/rotenone. (**A**) A graph of OCR values versus time-course (min). (**B**) Quantification of basal respiration OCRs. (**C**) Quantification of couple respiration OCRs (ATP production) (**D**) Quantification of maximal respiration capacity OCRs. (**E**) Quantification of proton leak respiration OCRs. (**F**) Quantification of ECARs. (**G**) The Western blot profile showed the effects of sinularin on the expression levels of complex I-NDUFB8, complex II-SDHB, complex III-UQCRC2, complex IV-COX II, and complex V-ATP5A. β-actin was used as the internal control. Full, uncropped Western blot gels are shown in Supplementary Figure S2D. The complex I-NDUFB8 (**H**), complex II-SDHB (**I**), complex III-UQCRC2 (**J**), complex IV-COX II (**K**), and complex V-ATP5A (**L**) protein levels were quantified by densitometry analysis using ImageJ software after being normalized with the β-actin level. Each bar represents the mean ± SE of three independent experiments. The results were analyzed using Student’s *t*-test to determine the significance, in which * *p* < 0.05 and ** *p* < 0.01 relative to the control (sinularin-untreated cells).

The oxygen consumption rate (OCR) of the mitochondria was measured following the sequential addition of inhibitors that specifically targeted the enzymatic complexes of the OXPHOS. The sequentially added inhibitors were oligomycin, which inhibits ATP synthase (also known as complex V), FCCP, which uncouples the OXPHOS, and rotenone, which inhibits complex I. Utilizing these inhibitors enabled us to derive the following mitochondrial respiration parameters: Basal respiration, ATP production (coupled respiration), maximal respiration, and proton leakage (uncoupled respiration). To understand the impact of sinularin on mitochondrial respiration, each respiration parameter was measured following treatment with sinularin at 0, 1, 5, and 10 μM for 4 h, quantified protein, and normalized respiration parameter in SK-HEP-1 cells (Figure 3A). After treatment with 10 μM of sinularin, our results demonstrated that basal mitochondrial respiration, ATP production, maximal respiration and proton leakage were significantly decreased to 130.08 ± 9.09, 63.33 ± 5.63, 287.08 ± 13.69 and 66.75 ± 4.53 pMoles/min/mg protein, relative to the control at 183.55 ± 13.58, 90.00 ± 8.35, 360.89 ± 12.84 and 93.56 ± 10.56 pMoles/min/mg protein, respectively (Figure 3B–E). A subsequent study was performed to measure the extracellular acidification rate (ECAR), which is an indicator of cellular glycolysis. Our findings demonstrated that 10 μM sinularin caused a significant decrease in ECAR to 65.75 ± 3.60 mpH/min/mg of protein, relative to the control (83.58 ± 4.38 mpH/min/mg of protein) (Figure 3F). We also monitored the expression levels of subunits of enzymatic complexes I to V protein using Western blot analyses by normalization -actin (Figure 3G). The subunit of complex I used for the Western blot analyses was NDUFB8, while the subunits for complex II, III, IV, and V were SDHB, UQCRC2, COX II, and ATP5A, respectively. Our results showed that the expression levels of all the tested subunits were significantly lowered after being treated with 10 μM of sinularin, to 0.16 ± 0.01, 0.43 ± 0.04, 0.29 ± 0.05, 048 ± 0.02, and 0.45 ± 0.01, relative to the control (normalization to 1), respectively (Figure 3H–L). These observations indicated that sinularin effectively decreased mitochondrial respiration, the glycolytic rate, and 5 OXPHOS complexes protein, causing mitochondrial dysfunction in SK-HEP-1 cells.

### 2.4. Sinularin Significantly Ablated Migration Ability and Colony Formation Potential

**Figure 4:**
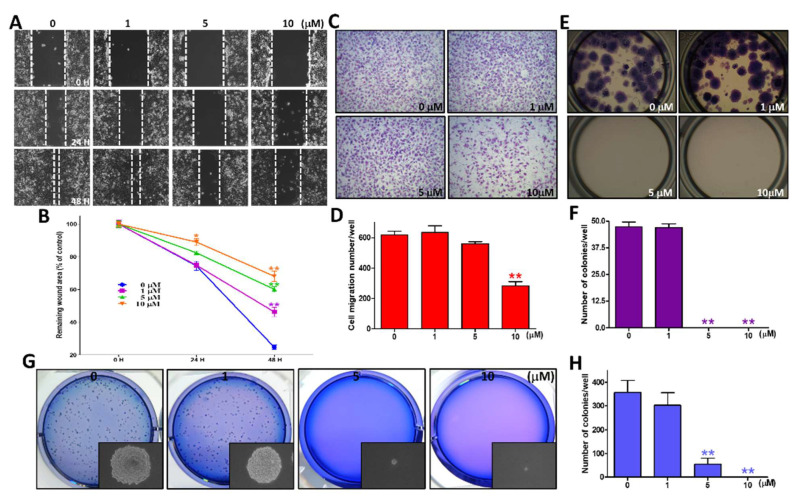
The effects of various concentrations of sinularin treatment on wound healing, cell migration, and colony formation of SK-HEP-1 cells. (**A**) The cells were scratched and treated with 0, 1, 5, and 10 μM of sinularin for 0, 24, and 48 h, and then photographed by phase-contrast microscopy at 100× magnification. (**B**) Statistics of the wound areas (blank indicates no cells) in the scratch-test assay, quantified using ImageJ software after being normalized with the time point 0 h at different concentrations of sinularin. (**C**) The profile of migration cells treated with 0, 1, 5, and 10 μM of sinularin for 4 h before being evaluated for chemotactic potency, captured by phase-contrast microscopy at 100× magnifications. (**D**) Quantification of the migration cell count using PhotoCapt software. (**E**) Colony formation morphology of attached cells. The cells were added to different concentrations of sinularin and incubated for 10 days, followed by fixation with 100% methanol and staining with 10% Giemsa stain. (**F**) Quantification of the number of colonies. (**G**) The soft agar colony formation assay was used to analyze the non-adherent growth ability of SK-HEP-1 cells after 14 days of co-culture with different concentrations of sinularin. A phase-contrast inverted microscope was used to photograph the pattern of cell colonies before staining, as shown in the under right corner of the frame. After staining with Giemsa stain, a digital camera was used to photograph the SK-HEP-1 colonies. (**H**) Quantification of the number of soft agar colonies. Each bar represents the mean ± SE of three independent experiments. The results were analyzed using Student’s *t*-test to determine the significance, in which * *p* < 0.05 and ** *p* < 0.01, relative to the control (sinularin-untreated cells).

Drug-induced inhibition of cell migration is an indicator that demonstrates if a drug has the potential to ablate cancer metastasis. We conducted a wound-healing assay and a transwell chamber migration assay, in which 0, 1, 5, and 10 μM of sinularin were applied. Following 48 h incubation of wound healing using the scratch assay, our findings showed that 1, 5, and 10 μM of sinularin could significantly abate the wound healing potential (Figure 4A,B). Following 4 h incubation in the transwell chamber migration assay, our results demonstrated that 10 μM of sinularin significantly decreased the migration ability of SK-HEP-1 cells to 282.25 ± 29.23, relative to the control, at 620.08 ± 23.46 (Figure 4C,D). We next evaluated the colony formation potentials of attached and non-attached SK-HEP-1 cells individually in separate experiments. For the attached cells, the colony formation potential was significantly decreased to a non-detectable level after being treated with 5 and 10 μM of sinularin, relative to the control, at 47.33 ± 2.02 (Figure 4E,F). For the non-attached cells, the colony formation potential was significantly decreased to a non-detectable level after being treated with various concentrations of sinularin, relative to the control, at 356.33 ± 50.80 (Figure 4G,H). These results suggested that the utilization of various concentrations of sinularin could significantly diminish cell migration and clonogenic formation, causing inhibition metastasis in SK-HEP-1 cells.

### 2.5. Sinularin Influenced the Distribution of Actin Filaments

**Figure 5:**
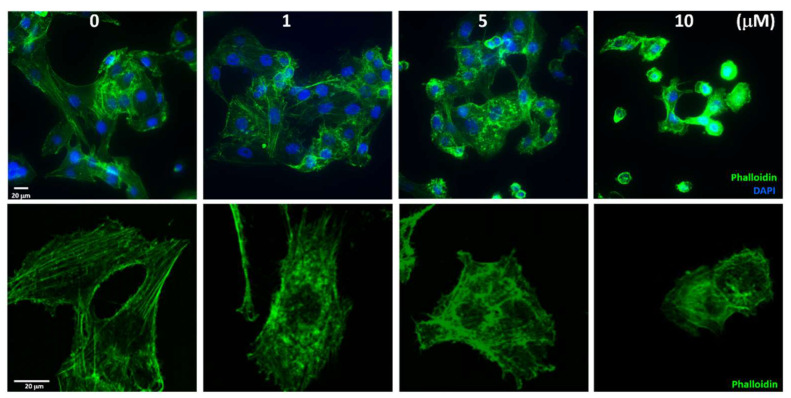
Sinularin effect F-actin expression in SK-Hep-1 cells. Immunofluorescence staining was used to evaluate the distribution of the F-actin filaments. The cells were pretreated with increasing concentrations of sinularin for 24 h and then stained with Alexa Fluor 488 Phalloidin (the F-actin filaments are shown in green) and DAPI (the nuclei position is shown in blue). The immunofluorescence profiles were visualized using a fluorescence microscope (upper, 400× magnification) and a confocal fluorescence microscope (lower, 1000× magnification).

Following the macroscopic observation of sinularin-induced cell migration, we next investigated microscopically using DAPI and phalloidin fluorescence dyes to track nuclei positions and F-actin filaments, respectively. Under no sinularin stress, the F-actin filaments tended to move away from the nuclei positions, showing filamentous extension morphology. Under 10 μM of sinularin, however, the filamentous extension contracted and became spheroid and remained nearby the nuclei positions, thus clearly showing cellular shrinkage and indicating apoptosis (Figure 5). The experimental fluorescence results indicated that sinularin could decrease the interference of F-actin filamentous on cell migration and promote cell apoptosis.

### 2.6. Sinularin Induces Apoptosis via MAPK, Epithelial-Mesenchymal Transition (EMT) and VEGF Signaling Pathways in SK-HEP-1 Cells

**Figure 6:**
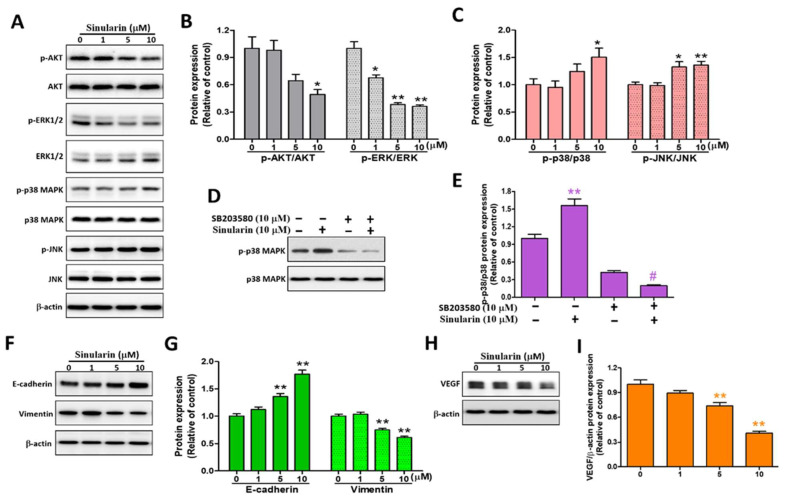
The effects of sinularin on AKT, MAPKs, E-cadherin, vimentin, and VEGF protein expression levels in SK-HEP-1 cells. SK-HEP-1 cells were treated with different concentrations of sinularin for 24 h. (**A**) The cell lysates were subjected to Western blot analyses with antibodies against p-AKT, AKT, p-ERK1/2, ERK1/2, p-p38 MAPK, p38 MAPK, p-JNK, and JNK and the internal control β-actin. Full, uncropped Western blot gels are shown in Supplementary Figure S3A. (**B**) p-AKT, AKT, p-ERK1/2, and ERK1/2 protein levels, quantified by densitometry analysis using ImageJ software after being normalized with the β-actin level. (**C**) p-p38 MAPK, p38 MAPK, p-JNK, and JNK levels, quantified by densitometry analysis using ImageJ software after being normalized with the β-actin level. (**D**) Pre-treatment with SB203580 reversed sinularin-induced p38 protein level. The cell lysates were subjected to Western blot analyses with antibodies against p-p38 MAPK and p38 MAPK. Full, uncropped Western blot gels are shown in Supplementary Figure S3B. (**E**) p-p38 MAPK and p38 MAPK protein levels, quantified by densitometry analysis using ImageJ software. (**F**) The cell lysates were subjected to Western blot analyses with antibodies against E-cadherin, vimentin, and β-actin. Full, uncropped Western blot gels are shown in Supplementary Figure S3C. (**G**) E-cadherin and vimentin protein levels, quantified by densitometry analysis using ImageJ software after being normalized with the β-actin level. (**H**) The cell lysates were subjected to Western blot analyses with an anti-VEGF antibody and β-actin. Full, uncropped Western blot gels are shown in Supplementary Figure S3D. (**I**) VEGF protein levels quantified by densitometry analysis using ImageJ software after being normalized with the β-actin level. Each bar represents the mean ± SE of three independent experiments. The results were analyzed using Student’s *t*-test and ANOVA to determine the significance, in which * *p* < 0.05, ** *p* < 0.01, and ^#^ *p* < 0.01, relative to the 10 μM sinularin-treated group.

AKT and mitogen-activated kinases (MAPKs), inclusive of ERK, p38, and JNK, have been reported to mediate cell migration and apoptosis [8,29], and we, therefore, analyzed their expression levels. The phosphorylated forms of the MAPKs (p-ERK, p-p38, and p-JNK) were also probed. After Western blot analyses, both the p-AKT/AKT and p-ERK/ERK ratios were significantly decreased to 0.49 ± 0.05 and 0.36 ± 0.02, while the p-p38/p38 and p-JNK/JNK ratios demonstrated a reverse result to 1.51 ± 0.16 and 1.36 ± 0.07, respectively, under 10 μM of sinularin, relative to the corresponding control (Figure 6A–C). Subsequently, the p38 inhibitor SB203580 was added, with and without 10 μM of sinularin. The results showed that SB203580 was able to significantly reverse the consequence of the sinularin-induced p-p38/p38 ratio (Figure 6D,E). It is known that maintaining the mesenchymal phenotype helps cell migration [9]. We next investigated the expression levels of E-cadherin and vimentin. Our findings showed that the E-cadherin was up-regulated, whereas the vimentin was down-regulated in a dose-dependent manner, with significant effects arising under 5 and 10 μM of sinularin (Figure 6F,G). Additionally, the VEGF expression level was also found to be decreased in a dose-dependent manner, with significant effects arising under 5 and 10 μM of sinularin (Figure 6H,I). The decrease in VEGF expression indicated that the angiogenesis potential was also decreased. These results demonstrated that the sinularin reduced SK-HEP-1 cell migration, cytoskeleton, and angiogenesis causing apoptosis via MAPK, EMT, and VEGF signaling pathways.

## 3. Discussion

In this study, we evaluated the anti-cancer effects of sinularin on SK-HEP-1 cells using an IC_50_ of ~10 µM. Our findings were consistent with the conclusion of a review article that found sinularin to be active against cancers [30]. In particular, our results were consistent with the results of Chung et al., although the HCC cell lines they used were HepG2 and Hep3B [25]. In the reported anti-cancer studies, the IC_50_ levels of sinularin in other cancer cell types were varied. Human HCC (HepG2) cells [25], HCC (Hep3B) cells [25], melanoma (A2058) cells [23], gastric cancer (AGS) cells [22], oral cancer (Ca9–22) cells [24], breast cancer (SKBR3) cells [21], renal cancer (786-O) cells [20], and renal cancer (ACHN) cells [20], respectively, showed the IC_50_ values of sinularin to be 17.5, 43.2, 9.28, 17.73, 23.5, 33, 124.4, and 132.5 µM after 24 h of incubation. This suggested that sensitivity to sinularin is cell type-dependent.

**Figure 7:**
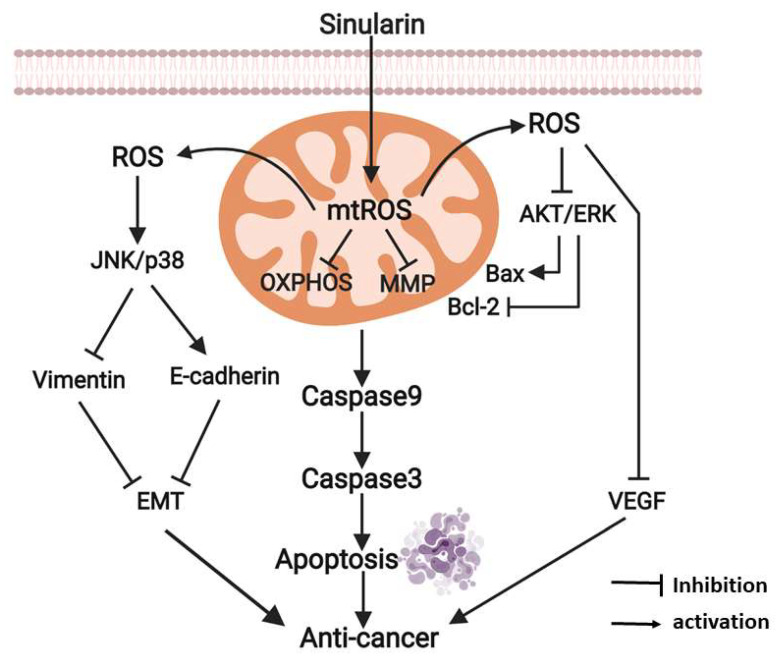
Proposed anti-cancer mechanisms for sinularin in SK-HEP-1 cells. The diagram was created with BioRender.com.

Following the understanding of the cytotoxic effects of sinularin, this compound was evidenced to induce mitochondria-associated anti-tumorigenesis signaling. The sinularin-induced anti-cancer mechanisms are proposed in Figure 7. As illustrated, sinularin inhibits the mitochondrion via the induction of mtROS, which is also induced by the blockade of MnSOD. The mtROS subsequently leads to the disruption of the OXPHOS in parallel with the disturbance to ΔΨ. The respiratory capacity of the mitochondrion is accordingly damaged. Such mitochondrial dysfunction results in the initiation of caspases 3/9 mediated apoptosis. Notably, significant disturbances to the mitochondrial membrane potentially stimulate additional ROS generation as a result of the disruption of the OXPHOS [31]. The elevated mtROS contributes to increasing intracellular ROS levels, which are unable to be normalized by Cu/ZnSOD as this superoxide dismutase is also ablated by sinularin. The resultant elevated intracellular ROS levels further attenuate AKT and ERK activities, which subsequently inhibit Bcl-2 and augment Bax. In addition, such ROS over-production inhibits VEGF while promoting the phosphorylation status of JNK and p38, leading to the down-regulation of vimentin and the up-regulation of E-cadherin. The changes in these two EMT regulators accordingly impact the EMT. Taken together, these inhibition effects result in anti-cancer effects that could effectively prevent hepatocellular cancer progression.

The anti-cancer effects induced by sinularin were considered to be a result of the activation of apoptosis and the inhibition of cell migration, metastasis, and angiogenesis. The apoptotic effect was first supported by cell cytometric analysis using Annexin V and PI staining, through which the late apoptotic cell population significantly appeared in the Annexin V+/PI+ quadrant under 10 μM of sinularin, compared to the control. DNA fragmentation was also observed using the TUNEL assay with DAPI dye. Further evidence demonstrated that the apoptosis effects were induced by mitochondria-associated apoptosis, which is an intrinsic cell death pathway regulated by caspases 9 and 3. The induction of mitochondria-associated apoptosis was subsequently found to be caused by elevated mtROS levels. Such increases in mtROS levels destructed the ΔΨ and mitochondrial respiratory capacity by the ablating activities of ETC complexes I to V. Additionally, the expression levels of MnSOD together with Cu/ZnSOD were attenuated due to increased intracellular ROS levels. Further probing found that the sinularin also elicited anti-cell migration and anti-metastasis effects. Supported by the wound healing assay and transwell migration assay, sinularin was found to lower the cell migration potential. It has been reported that the acquisition of the mesenchymal phenotype through the EMT promotes the migration potential, thus increasing the likelihood of metastasis [9]. Accordingly, soft agar assays were performed, and EMT biomarkers were monitored. Sinularin was found to decrease the colony formation capacity of the HCC cells. In addition, the vimentin level was attenuated, whereas the amount of E-cadherin was increased. Such an anti-migration effect was further visualized in situ using confocal microscopy, which showed cellular shrinkage caused by the filamentous contraction. Further investigation found that the angiogenesis potential was also attenuated by sinularin through the down-regulation of the VEGF expression level. Overall, our findings supported sinularin-induced anti-cancer effects.

The over-production of mtROS under sinularin treatment is considered a critical factor that induces anti-cancer effects. This sinularin-induced ROS over-production effect may bring it an advantage. It has been long known that a hypoxic tumor microenvironment is an impediment to cancer therapies. The induction of mtROS could be an effective approach to alleviating hypoxia [32]. By inducing ROS levels in the mitochondria, it is expected that the oxygen consumption of the OXPHOS will decline, consequently resulting in an increase in oxygen levels in the cytosol and surrounding tissues. Sinularin shows strong potential for the increase of oxygen availability to the cytosol and tissues, as it significantly attenuates respiration capacity at its IC_50_ value. Accordingly, sinularin could be a useful tool to study the hypoxic tumor microenvironment.

## 4. Materials and Methods

### 4.1. Compound and Reagents

The marine-derived compound sinularin (5,15-dioxatricyclo [12.3.1.0(4,6)] octadec-9-en-16-one) was isolated from soft coral and provided by Professor Jyh-Horng Sheu (Department of Marine Biotechnology and Resources, National Sun Yat-sen University, Taiwan) and Professor Ping-Jyun Sung (National Museum of Marine Biology and Aquarium, Pingtung, Taiwan). A stock solution of sinularin was prepared in dimethyl sulfoxide (DMSO), protected from light, and stored at −20 °C. Phosphate-buffered saline (PBS) was bought from GMbiolab Co Ltd. (Taichung, Taiwan). An Annexin V-PI kit, MitoSOX^TM^ Red, the chloromethyl derivative of 2,7-dichlorodihydro- fluorescein diacetate (CM-H_2_DCFDA), Rhodamine123, JC-1, DAPI (4′,6-Diamidino-2-Phenylindole, Dilactate), and 488-phalloidin were purchased from Molecular Probes, Inc. (Eugene, OR, USA). MitoTempo, DMSO, SB203580, and 3-(4,5)-dimethylthiazol(-z-y1)-3,5-diphenyltetrazolium bromide (MTT) were purchased from Sigma-Aldrich Corporation (St. Louis, MO, USA). Before use, SB203580 and MTT were dissolved in DMSO at a concentration of 10 mM and 5 mg/mL as stock solutions. In Situ Cell Death Detection Kit, Fluorescein for the Terminal Transferase-mediated dUTP Nick-End Labeling (TUNEL) assay was purchased from Roche Life Science (Penzberg, Upper Bavaria, Germany). The caspase-3 inhibitor [Z-Asp(OMe)-Glu-Val-Asp(OMe)-fluoromethyl ketone (Z-AEVD-FMK)] was purchased from Sigma-Aldrich (St. Louis, MO, USA). The caspase 3 (cleaved) Human ELISA and Human VEGF DuoSet ELISA kit assay were purchased from Thermo Fisher Scientific (Waltham, MA, USA) and R & D Systems, Inc. (Minneapolis, MN, USA). A Seahorse XF Cell Mito Stress Test kit, including oligomycin, carbonyl cyanide-4-(trifluoromethoxy) phenylhydrazone (FCCP), and rotenone were purchased from Agilent Technologies, Inc. (Santa Clara, CA, USA).

### 4.2. Cell Culture

SK-HEP-1 cells (ATCC^®^ HTB-52^TM^, liver adenocarcinoma cells of Homo sapiens) and clone 9 cells (ATCC^®^ CRL-1439^TM^, normal liver cells of rat) were purchased from American Type Culture Collection (Manassas, VA, USA). Furthermore, Huh-7 cells (hepatocellular carcinoma cells of Homo sapiens) were kindly provided by Professor Ming-Hong Tai (National Sun Yat-sen University). SK-HEP-1 and Huh-7 cells were cultured in DMEM medium (Gibco BRL, Rockville, MD, USA), and clone 9 cells were cultured in F-12K medium (Gibco) containing 10% fetal bovine serum, 2 mM glutamine, 100 U/mL penicillin, and 100 µg/mL streptomycin (Gibco) under a humidified atmosphere of 5% CO_2_ and 95% room air at 37 °C. For subculture, the cells were treated with trypsin-EDTA (Gibco).

### 4.3. Cell Viability Assay

The viability of SK-HEP-1, Huh-7, and clone 9 cells after treatments with sinularin at 0, 0.5, 1, 5, 10, 25, 50, and 100 μΜ for 24, 48, and 72 h were evaluated using the MTT assay in triplicate. In brief, the cells were plated in 96-well microplates (Nunc™ MicroWell™, Thermo Fisher Scientific, Waltham, MA, USA) at a density of 5 × 10^3^ cells per well. Following overnight incubation, the cells were treated with the indicated concentrations of sinularin for 24, 48, and 72 h. Subsequently, 20 µL of 5 mg/mL MTT solutions were added to wells, followed by incubating at 37 °C for 3 h. The absorbance from the resulting reduced product of MTT by viable cells was recorded at 570 nm using a microplate reader (Dynatech Laboratories, Chantilly, VA, USA). The relative cell viability (expressed in %) was calculated as the optical density of sinularin-treated cells divided by the optical density of untreated control cells and multiplied by 100. The percentage of viable cells was expressed as the mean ± standard error (SE).

### 4.4. Annexin V-FITC/PI Staining

SK-HEP-1 cells were treated with sinularin at 0, 1, 5, and 10 μΜ for 24 h, then harvested, washed twice with PBS, and centrifuged. The 100 mL of the solution (1 × 10^5^ cells) were then transferred into a tube. Samples were processed for Annexin V and propidium iodide (PI) label as per the manufacturer’s manual (BD Biosciences, East Rutherford, NJ, USA). The cells were first resuspended in a binding buffer and then labeled with fluorescence to detect early apoptotic, late apoptotic, and necrotic cells by adding 5 μL of Annexin V-FITC and 5 μL of PI to each sample. Samples were gently vortexed and placed at room temperature in the dark for 10 min. Upon completion of the incubation, 400 μL of 1X binding buffer was added to each sample, and the samples were analyzed using a Beckman CytoFLEX flow cytometer (Southfield, MI, USA) to detect the fluorescence intensity of annexin V (green fluorescence)/PI (red fluorescence) and with the use of CytExpert 2.0 analysis software. A minimum of 20,000 cells per sample was analyzed.

### 4.5. Terminal Transferase-Mediated dUTP Nick-End Labeling (TUNEL) Assay

The TUNEL assay was used to detect late stages of apoptosis and DNA fragmentation. SK-HEP-1 cells were seeded on cover slides with overnight incubation, followed by sinularin treatment at 0, 1, 5, and 10 μΜ for 24 h. Next, the cells were fixed with 4% neutral formalin (pH = 7.2) for 10 min at 4 °C and blocked with 1% BSA solution for 30 min. The staining was performed using the In Situ Cell Death Detection Kit, Fluorescein, according to the manufacturer’s protocol (Roche Life Science). Subsequently, these cells were stained with DAPI (for detecting nuclei positions) for 10 min at room temperature, washed with PBS, and covered with a seal sheet. Under fluorescence microscopy (Leica Microsystems; Wetzlar, Germany) was visualized, TUNEL positive cells emitted in green fluorescence. Those cells showing TUNEL (green fluorescence) were indicated by co-localization with DAPI (blue fluorescence) and by cellular nuclear morphology. A fluorescence microscope was used with green fluorescence set at 488 nm and blue fluorescence set at 405 nm to visualize the slides. A SPOT CCD RT-slider integrating camera (Diagnostic Instruments, Sterling Heights, MI, USA) was used to capture the images. The cells emitting green fluorescence indicated apoptotic cells, while blue indicated nuclei positions.

### 4.6. Western Blot Analysis

SK-HEP-1 cells were treated with sinularin for 24 h, followed by the harvest of both adherent and floating cells. The cells were subjected to ice-cold PBS wash, added RIPA lysis buffer (Sigma-Aldrich Corporation) for 2 h, and centrifuged at 13,500 rpm for 30 min at 4 °C to acquire the supernatant. The resulting proteins were subjected to a detergent compatible (DC) assay (Bio-Rad Laboratories Inc., Hercules, CA, USA) to determine protein concentrations. After the extract of 20–50 μg proteins taken from each group and then separation using 8%–15% sodium dodecyl sulfate polyacrylamide gel electrophoresis was completed, cellular proteins were transferred onto polyvinylidene fluoride membranes (EMD Millipore Corporation, Billerica, MA, USA), blocked with 5% milk in Tris-buffered saline (GMbiolab Co Ltd., Taichung, Taiwan) and Tween 20 (Sigma-Aldrich, St. Louis, MO, USA) for 1 h. Subsequently, the membranes were incubated with primary antibodies against the desired proteins at 4 °C, of which antibodies included cell survival-related (Bax, Bcl-2), mitochondrial bioenergetics-related proteins (Complexes I to V), ROS-scavenge related proteins (superoxide dismutases MnSOD, Cu/ZnSOD), MAPK kinases (AKT, ERK, p38, JNK), E-cadherin, vimentin, VEGF, and β-actin (housekeeper protein) as shown in Table S1. The following step was to conjugate with horseradish peroxidase (HRP) secondary antibody for 1 h. The bands of immobilized proteins were then visualized using an enhanced chemiluminescence kit (Millipore Corporation, Billerica, MA, USA). BioSpectrum Imaging System (Analytik Jena US LLC, Upland, CA, USA) was used to capture the images of the bands. LabWorks 4.0 software (Analytik Jena US LLC) was utilized to conduct relative densitometry quantification of the immune-reactive bands. The β-actin antibody was used as a loading control. For the cells co-treated sinularin with the caspase-3 inhibitor Z-DVED-FMK, the same procedure as described above was followed.

### 4.7. Measurement of Cleaved Caspase-3 and VEGF by ELISA Kit

Levels of cleaved caspase-3 and VEGF in the SK-HEP-1 cells were measured using an enzyme-linked immunosorbent assay (ELISA). The human cleaved-caspase-3 ELISA kit (KHO1091) and human VEGF ELISA kit (DY293B) were used as per the manufacturer’s protocol. The total protein quantity of each sample was quantified using a DC protein assay.

### 4.8. Measurement of Intracellular and Mitochondrial ROS (Reactive Oxygen Species) Levels

The measurements of intracellular and mitochondrial ROS levels were conducted following the publication of Yuan et al. [13]. Intracellular ROS levels were evaluated using the probe 2′,7′-dichlorodihydroflorescein diacetate (CM-H_2_DCFDA). This probe was accumulated by cells and converted into 2′,7′-dichloroflorescein (DCF) in the presence of cellular ROS. This product was detected at an emission wavelength of 530 nm (excitation wavelength of 485 nm). Briefly, 3 × 10^5^ cells were plated in 6-well plates and allowed to attach for 16–18 h. The attached cells were treated with sinularin at 0, 1, 5, and 10 μΜ for 24 h, followed by incubating with CM-H_2_DCFDA (5 μM) for an additional 30 min at 37 °C, then washed and resuspended in PBS. Beckman CytoFLEX flow cytometer was utilized to measure the intracellular ROS level. Data were analyzed using the flow cytometry analysis software CytExpert 2.0. For determining the mitochondrial ROS level, MitoSOX^TM^ Red kit was used. MitoSOX^TM^ Red can detect the level of mitochondrial superoxide (O^2−^) production. Briefly, the treated cells were incubated with 5 μM MitoSOX^TM^ Red in PBS for 30 min at 37 °C, then washed and resuspended in 0.5 mL of PBS, followed by being subjected to flow cytometry. A minimum of 20,000 cells per sample was analyzed.

### 4.9. Measurement of Mitochondrial Membrane Potential (ΔΨ) by Flow Cytometry

The measurement of mitochondrial membrane potential (ΔΨ) was modified from the literature [33]. Briefly, SK-HEP-1 cells were seeded at a density of 3 × 10^5^ cells/well in 6-well plates and allowed to attach for 16–18 h. Afterward, the cells were treated with sinularin at 0, 1, 5, or 10 μΜ for 8 h and washed with PBS. The fluorescent dyes (Rhodamine 123 or JC-1) were added with proper volumes into the Hanks’ Balanced Salt solution (HBSS), and the solution was incubated at 37 °C for 20 min. The medium was then removed, and trypsin was added to the cells. Subsequently, the cells were harvested and resuspended in 1 mL of HBSS. The CytoFLEX (Beckman Counter) was used to detect the fluorescence intensity of low and high mitochondrial membrane potentials. At least 20,000 cells/group were analyzed using CytExpert 2.0.

### 4.10. Mitochondrial Function Measurements

The mitochondrial function measurements were modified from the literature [34]. Oxygen consumption in cells and mitochondria was determined using the Seahorse XF24 Extracellular Flux Analyzer (Seahorse Bioscience, Chicopee, MA, USA). To allow comparisons between experiments, data were presented as oxygen consumption rate (OCR) in pmol/min/mg protein and extracellular acidification rate (ECAR) in mpH/min/mg protein cells. Cells were seeded in triplicate at a density of 7 × 10^4^ cells per well in seahorse cell culture 24-well Seahorse XF microplates. After overnight incubation, the cells were subjected to DMEM medium and 10% FBS containing various concentrations of sinularin for 4 h. After washing the cells with 1 mL of Seahorse media (DMEM without sodium bicarbonate, 37 °C, pH = 7.4), 675 mL of the seahorse medium were added. Basal OCR was measured 4 times and plotted as a function of cells under the basal condition followed by the use of Seahorse XF Cell Mito Stress Test kit (Seahorse Bioscience Inc., Chicopee, MA, USA)) and sequential addition of oligomycin (1 mM), FCCP (0.5 mM) as well as rotenone/antimycin (1 mM). At the end of the recording period, the cells were collected, and protein quantification was determined using a DC assay, as standardized values of OCR and ECAR were calculated.

### 4.11. Wound Healing Measurement (Scratch-Test Assay)

The wound healing measurement (scratch-test assay) procedure was modified from Tseng et al. [35]. The pre-cultured SK-HEP-1 cells were seeded in 24-well plates (50,000 cells per well) as a monolayer. A straight line was made with a 10 μL tip and ruler. A scratch across the cell monolayer was formed at the bottom of each 24-well dish, creating a field without any cells, and the edge of the field was smoothened. Subsequently, PBS was used to wash the cells, followed by the addition of 0, 1, 5, or 10 μM of sinularin, and the cells were further cultured for an additional 48 h. At 0, 24, and 48 h, the images were photographed using an inverted phase-contrast microscope (Leica Microsystems GmbH, Wetzlar, Germany), and the same wound locations were observed and recorded. ImageJ analysis software (National Institutes of Health, Bethesda, MD, USA) was used to analyze the “wound” areas (no cells area) and to compare the remaining “wound” areas between the control groups.

### 4.12. Transwell Migration Chamber Assay

The procedure of transwell migration chamber assay was referenced from the publication of Chen et al. [34]. 24-transwell (#3422, BD FalconTM Cell Culture Inserts, BD Biosciences, Bedford, MA, USA) inserts with 8 µm pore size were selected. In brief, SK-HEP-1 cells were treated with trypsin to form a resuspension on top chamber of the filter membrane, at a density of 2 × 10^4^ per well in 1% FBS supplemented culture medium containing 0, 1, 5, and 10 μM concentrations sinularin. The chemo-attractant at the lower chamber was 10% FBS to induce cell migration. Following 16 h incubation, residual solution and non-migrated cells from the upper layer of the membrane were removed using cotton-tipped applicators with care. The migrated cells on the other side of the membrane were washed with PBS, fixed with 4% formaldehyde, and Giemsa stained for 30 min. A phase-contrast microscope was utilized for observations and recordings of the lower part of the membrane, and the images were captured using a SPOT RT Slider CCD Scientific Digital Camera System (Diagnostic Instruments, Sterling Heights, MI, USA). In the transwell migration images, ImageJ software was used to analyze the counter number of migrated cells in every field and 3 randomly selected regions on every transwell chamber insert membrane.

### 4.13. Colony Formation Assay

SK-HEP-1 cells were cultured at a density of 1000 cells/well in the culture medium supplemented with 10% FBS (Fetal Bovine Serum) and 1% antibiotics in a 24-well plate incubated in a CO_2_ incubator at 37 °C and 5% CO_2_. Afterward, the cells were treated with sinularin at 0, 1, 5, or 10 μΜ for 14 days. The culture medium and sinularin treatments were replaced with fresh medium/treatments every 2 days. Upon completion, the cells were washed with PBS, fixed with 4% formaldehyde, and stained with 10% Giemsa for 30 min. A phase-contrast microscope (Z16 APO, Leica, Wetzlar, Germany) was utilized for observations and recordings of the colony formation and its morphology. The colony cells (>250 cells per colony) were analyzed quantitatively using the computing PhotoCap 5.0 software.

### 4.14. Soft Agar Colony Formation Assay

The cells were cultured in semi-solid low-concentration agarose to observe the non-adherent growth ability of the cells in a suspension environment. First, 0.6% low melting point agar colloid containing normal cell growth medium (10% FBS) in a 6-well cell culture dish was solidified to be the lower layer. Afterward, 0.3% low melting point agar colloid agar containing normal cell growth medium was used as the upper layer. The 0.3% agar was mixed with the 1000 cells/well along with different concentrations of sinularin, and then laid flat on top of the solidified lower layer. After the upper layer of colloid became solidified, the plates were incubated in a CO_2_ incubator at 37 °C and 5% CO_2_ for 14 days. Before staining, a phase-contrast inverted microscope (Leica DMI 3000B) was used to photograph the shape of a single colony morphology. After staining with 10% Giemsa stain, a digital camera (Coolpix P6000, Nikon, Tokyo, Japan) was used to take a picture of the colony formation in the cell culture plate. The colony cells (>250 cells per colony) were analyzed quantitatively using the computing PhotoCap 5.0 software.

### 4.15. Confocal Microscopy for Immunofluorescence Phalloidin Staining of Cells

Firstly, 1.2 × 10^5^ SK-HEP-1 cells were seeded on coverslips, followed by the addition of 0, 1, 5, or 10 μM sinularin into the medium and incubated overnight. The cells were fixed with 4% formalin–PBS for 10 min. The cells were then washed 3 times with PBS buffer and incubated with blocking solution containing 4% goat serum. After blocking, the specific antibody for F-actin, Alexa Fluor 488 Phalloidin (green), was incubated for 30 min at room temperature before PBS wash to remove excessive solutions. Subsequently, these cells were stained with DAPI for 10 min at room temperature, washed with PBS, and covered with a seal sheet. For immunostaining analysis, the stained sections were examined under a Leica TCS SP II confocal microscope (Leica Instruments Inc., Wetzlar, Germany). Those cells showing F-actin (green fluorescence) were indicated by co-localization with DAPI (blue fluorescence) and by cellular/nuclear morphology. A fluorescence microscope was used with green fluorescence set at 488 nm and blue fluorescence set at 405 nm to visualize the slides. A SPOT CCD RT-slider integrating camera (Diagnostic Instruments, Sterling Heights, MI, USA) was used to capture the images. The cells emitting green fluorescence indicated apoptotic cells, while blue indicated nuclei positions.

### 4.16. Statistical Analysis

SPSS software (Windows 13.0 version; SPSS Inc., Chicago, IL, USA) was used for all statistical analyses. IC_50_ values were determined using a four-parameter logistic function. Results were analyzed using the t-test and presented as mean ± SE. * *p* < 0.01 or ** *p* < 0.05 are considered to be statistically significant.

## 5. Conclusions

Sinularin showed cytotoxicity selectivity over HCC cells and induced mitochondria-modulated apoptosis and cytoskeleton disruption.

## Supplementary Materials

The following are available online at https://www.mdpi.com/article/10.3390/ijms22083946/s1, Table S1: Information on primary and secondary antibodies used in the western blot analysis of this study. Figure S1: The effects of sinularin on cell viability in Huh-7 and Clone 9 cells:, Figure S2: Original, uncropped images of the western blots for Fig. 1H, 2E, 2K and 3G displayed in the text and results, Figure S3: Original, uncropped images of the western blots for Fig. 6A, 6D, 6F, and 6H displayed in the text and results.
