# Supplementary material for: Sinularin, an Anti-Cancer Agent Causing Mitochondria-Modulated Apoptosis and Cytoskeleton Disruption in Human Hepatocellular Carcinoma
Source: Int J Mol Sci. 2021 Apr 11;22(8):3946. doi: 10.3390/ijms22083946 (PMC8069418; doi:10.3390/ijms22083946)
Supplement: Supplementary file 1 [file ijms-22-03946-s001.pdf]

Sinularin, an anti-cancer agent causing mitochondria-modulated apoptosis and cytoskeleton disruption in human hepatocellular carcinoma

Chou-Yuan Ko [1,2†], Po-Chang Shih [3,4†], Po-Wei Huang [3], Yi-Hsin Lee [3], Yen-Fu Chen [1], Ming-Hong Tai [5,6], Chi-Hao Liu [7], Zhi-Hong Wen [3,*], Hsiao-Mei Kuo [3,6,*]

[1]Division of Gastroenterology and Hepatology, Department of Internal Medicine, Kaohsiung Armed Forces General Hospital, Kaohsiung, 80284, Taiwan; gastroenterokjy@gmail.com (C.-Y.K.); lineageofray@hotmail.com (Y.-F.C.)

[2] Institute of Medical Science and Technology, National Sun Yat-Sen University, Kaohsiung, 80424, Taiwan

[3]Department of Marine Biotechnology and Resources, National Sun Yat-sen University, Kaohsiung, 80424, Taiwan; smoke88722@gmail.com (P,-W.H.); po-chang.shih.14@ucl.ac.uk (P.-C.S.); lesinsin@gmail.com (Y.-H.L.); Hsiaomeikuo@g-mail.nsysu.edu.tw (H.-M.K.)

[4] UCL School of Pharmacy, University College London, Bloomsbury, London WC1N 1AX, UK

[5] Institute of Biomedical Sciences, National Sun Yat-sen University, Kaohsiung 80424, Taiwan; minghongtai@gmail.com (M.-H.T.)

[6]Center for Neuroscience, National Sun Yat-sen University, Kaohsiung 80424, Taiwan; minghong-tai@gmail.com

[7]Division of Nephrology, Department of Interanl Medicine, Kashsiung Armed Forces General Hospital, Kaohsiung 80284, Taiwan; colinliu1201@gmail.com

*Correspondence: wzh@mail.nsysu.edu.tw (Z.-H.W.); Hsiaomeikuo@g-mail.nsysu.edu.tw (H.-M.K.);

Tel.: +886-7-5252000 (ext. 5038) (Z.-H.W. & H.-M.K.); Fax: +886-7-5252021 (Z.-H.W. & H.-M.K.)

[†]These authors contributed equally to this work.

1. Supplementary Table

Supplementary Table S1. Information on primary and secondary antibodies used in the western blot analysis of this study.

Primary Antibody	Catalogue	Secondary antibody	Provider	Dilution
Caspase-9	9502	Mouse	Cell Signaling	1:1000
Pro-caspase-3	9662	Rabbit	Cell Signaling	1:500
Cleaved-caspase-3	9664	Rabbit	Cell Signaling	1:500
Cu/Zn SOD	ab13498	Rabbit	Abcam	1:500
Mn SOD	sc-133134	Mouse	Santa Cruz	1:1000
Bcl-2	15071	Mouse	Cell Signaling	1:1000
Bax	2772S	Rabbit	Cell Signaling	1:1000
Total OXPHOS Human WB Antibody Cocktail	ab110411	Mouse	Abcam	1:1000
p-AKT	9271s	Rabbit	Cell Signaling	1:1000
AKT	9272S	Rabbit	Cell Signaling	1:1000
p-ERK1/2	9101S	Rabbit	Cell Signaling	1:1000
ERK1/2	9102S	Rabbit	Cell Signaling	1:1000
p-p38 MAPK	45115	Rabiit	Cell Signaling	1:1000
p38 MAPK	9212	Rabbit	Cell Signaling	1:1000
p-JNK	4668s	Rabbit	Cell Signaling	1:1000
JNK	9252s	Rabbit	Cell Signaling	1:1000
E-cadherin	ab53033	Rabbit	Abcam	1:1000
vimentin	ab45939	Rabbit	Abcam	1:1000
VEGF	05-443	Mouse	Millipore	1:1500
β-actin	A5441	Mouse	Sigma-Aldrich	1:5000

2. Supplementary Figures

A

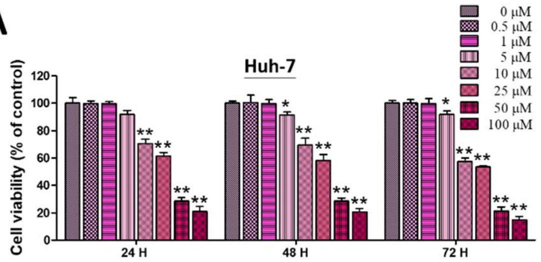

B

Time (hours)	IC_{50} (µM)	R^2	95% Confidence Interval
**24**	19.33	0.96	18.7 to 19.9
**48**	18.09	0.96	17.5 to 18.7
**72**	17.20	0.91	16.6 to 17.8

C

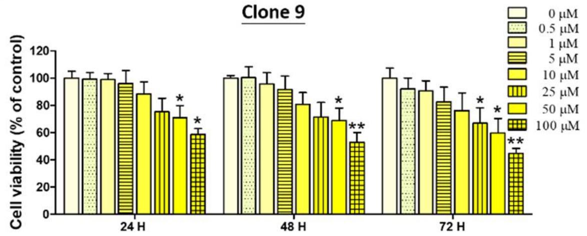

D

IC_{50} of sinularin for cell viability	
**Time (hours)**	**Clone 9 (µM)**
**24**	>100
**48**	>100
**72**	>100

Supplementary Figure S1. The effects of sinularin on cell viability in Huh-7 and Clone 9 cells: (**A**) Quantification of Huh-7 cell viability using the MTT assay with sinularin at concentrations of 0, 0.5, 1, 5, 10, 20, 50 or 100 µM after 24, 48 and 72 h, with results expressed as the percentage of viable cells compared to untreated cells (0 µM); (**B**) Determination of IC_{50}, R^2, and 95% confidence interval values for sinularin in Huh-7 cells after 24, 48 and 72 h; (**C**) Quantification of Clone 9 cell viability using the MTT assay with sinularin at concentrations of 0, 0.5, 1, 5, 10, 20, 50 or 100 µM after 24, 48 and 72 h, with results expressed as the percentage of viable cells compared to untreated cells (0 µM); (**D**) Determination of IC_{50} for sinularin in Clone 9 cells after 24, 48 and 72 h. * $p < 0.05$ and ** $p < 0.01$ relative to the control (sinularin-untreated cells).

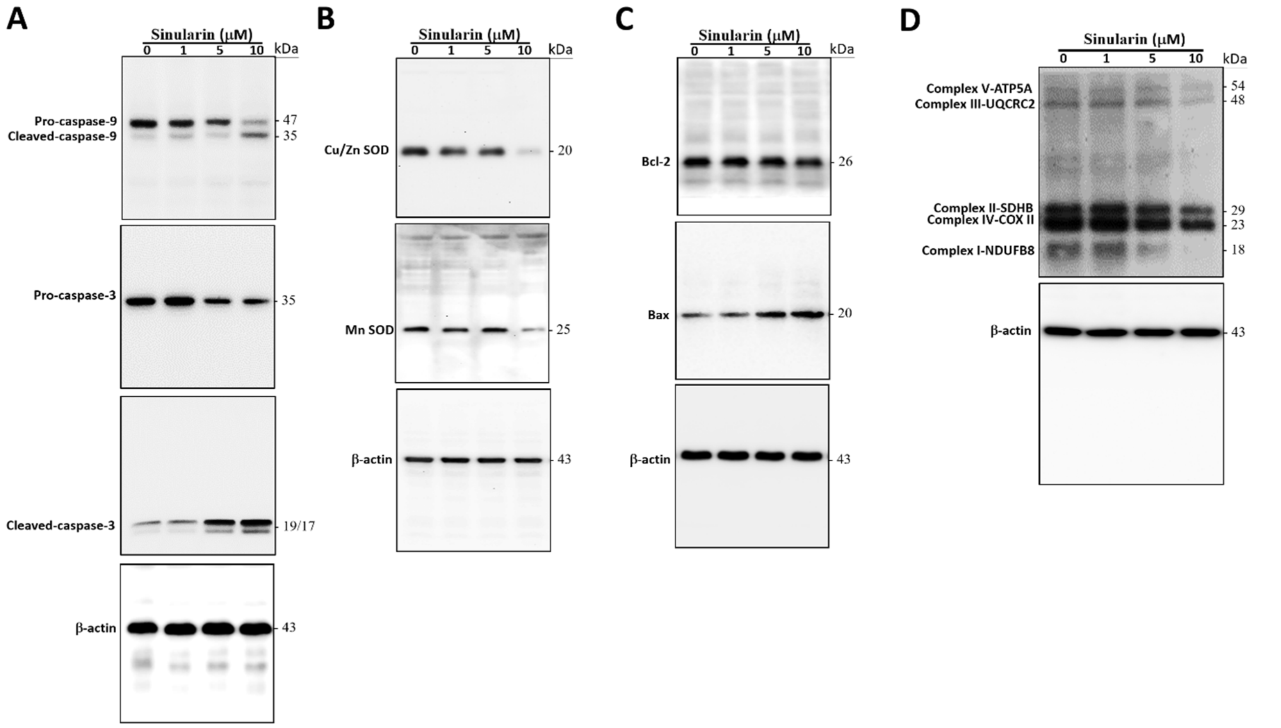

Supplementary Figure S2. Original, uncropped images of the western blots for Fig. 1H, 2E, 2K and 3G displayed in the text and results: (**A**) The bands of the cleaved caspase-9, pro-caspase-9, cleaved caspase-3, pro-caspase-3 and β-actin and their expected molecular weight, with β-actin used as the protein loading control; (**B**) The bands of Cu/Zn SOD, Mn SOD and β-actin and their expected molecular weight, with β-actin used as the protein loading control; (**C**) The bands of Bcl-2, Bax, and β-actin bands and their expected molecular weight, with β-actin used as the protein loading control; (**D**) The bands of indicated OXPHOS enzymatic complexes examined using Total OXPHOS Human WB Antibody Cocktail (Abcam, Cambridge, UK), with β-actin used as the protein loading control.

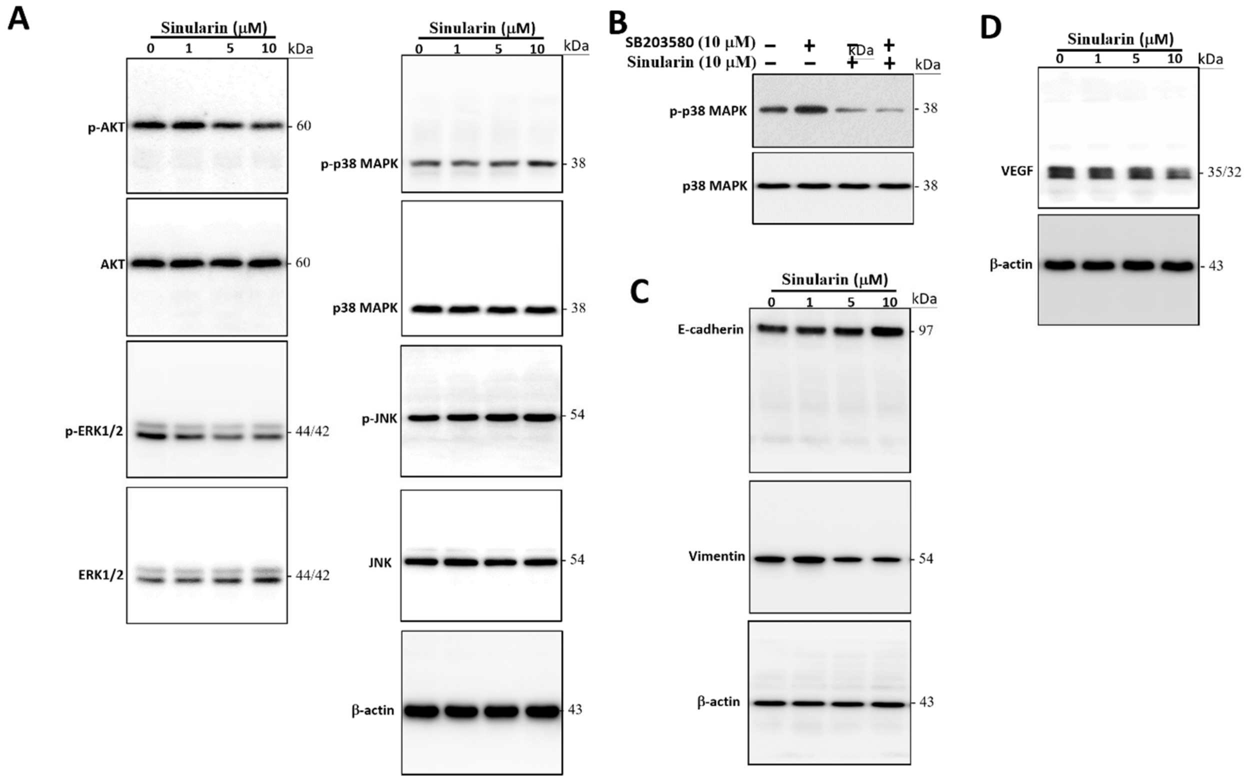

Supplementary Figure S3. Original, uncropped images of the western blots for Fig. 6A, 6D, 6F, and 6H displayed in the text and results: (**A**) The bands of p-AKT, AKT, p-ERK1/2, ERK1/2, p-p38 MAPK, p38 MAPK, p-JNK, JNK and β-actin and their expected molecular weight, with β-actin used as the protein loading control; (**B**) The bands of p-p38 MAPK and p38 MAPK and their expected molecular weight; (**C**) The bands of E-cadherin, vimentin and β-actin and their expected molecular weight, with β-actin used as the protein loading control; (**D**) The bands of VEGF and β-actin and their expected molecular weight, with β-actin used as the protein loading control.
